# Beliefs regarding medication and side effects influence treatment adherence in adolescents with attention deficit hyperactivity disorder

**DOI:** 10.1007/s00787-016-0919-1

**Published:** 2016-11-15

**Authors:** Maria Emilsson, Per A. Gustafsson, Gisela Öhnström, Ina Marteinsdottir

**Affiliations:** 1 Department of Health Science, Section of Nursing Graduate Level, University Wes, 461 86 Trollhättan, Sweden; 2 Department of Clinical and Experimental Medicine and Department of Child and Adolescent Psychiatry, Center for Social and Affective Neuroscience, Linköping University, 581 85 Linköping, Sweden; 3 Department of Clinical and Experimental Medicine, Center for Social and Affective Neuroscience, Linköping University, 581 85 Linköping, Sweden

**Keywords:** ADHD, Adolescents, Medication beliefs, Perception, Treatment adherence

## Abstract

Adherence to attention deficit hyperactivity disorder (ADHD) treatment is important because, when untreated, it may have serious consequences with lifelong effects. In the case of adolescents on long-term medicine prescription, more knowledge is needed regarding adherence and factors influencing adherence, which was the purpose of this study. Adolescents (*n* = 101) on ADHD medication ≥6 months were administrated questionnaires at a monitoring appointment: Medication Adherence Report Scale (MARS), beliefs about medicines (BMQ) and the Brief Illness Perception Questionnaire (B-IPQ). Adherence was high, the mean value was 88% of the maximum MARS score, and correlated positively with the “BMQ-necessity-concerns differential” but negatively with “BMQ-concerns” and “BMQ-side effects”. Adolescents with more belief in the necessity of the medication, less concerns and less experience of side effects tended to be more adherent to medication prescription (“intentional non-adherence”), while “unintentional non-adherence” (forgetfulness) was associated with how much they perceived that their ADHD affected their lives. In a multiple regression model, the variance of MARS total (*R* ^2^ = 0.21) and “intentional non-adherence” (*R* ^2^ = 0.24) was explained by the “BMQ-necessity–concern differential” and “BMQ-experienced side effects”. The variance of “unintentional non-adherence” (*R* ^2^ = 0.12) was explained by the “BMQ-necessity–concern differential” and “B-IPQ-consequences of ADHD”. In conclusion, adolescents on long-term medication reported good adherence, mainly influenced by more beliefs in the necessity versus concerns of the medications, less experienced side effects and more perceived consequences of ADHD. BMQ could be useful to identify risks of low adherence, which should be counteracted by partially gender-specific interventions.

**Electronic supplementary material:**

The online version of this article (doi:10.1007/s00787-016-0919-1) contains supplementary material, which is available to authorized users.

## Introduction

Attention deficit hyperactivity disorder (ADHD) is a neuropsychiatric disorder found in about 7% of children [1] and in approximately 3.4% of adults [2]. ADHD is treated primarily by medications such as methylphenidate (MPH), amphetamine and atomoxetine (ATX), in combination with psychoeducational measures and support [3, 4]. Stimulants like MPH lead to a more general increase of dopamine in the brain, while ATX increases norepinephrine selectively and dopamine indirectly in the frontal cortex [5]. Adolescence is a vulnerable period and the expression of ADHD during this period may have destructive effects on the entire life course, resulting in fewer friends, school difficulties and dismissals, arrests, unwanted pregnancy, sexually transmitted diseases and abuse of drugs or alcohol [6–8]. However, medical treatment for individuals with ADHD is reported to reduce the risk of substance abuse [9], comorbid psychiatric disorders [10] and criminality [11]. In general, adherence is important in achieving treatment effects [12]. For instance, high adherence to ADHD medication in school settings has been shown to yield higher academic grades among young people with ADHD [13].

Adolescents in the process of detachment from parental supervision might especially be expected to be less adherent [14], at least compared with younger children under parental supervision [15–17]. Available evidence shows adherence failure in about 50% of children and adolescents [18]. On the other hand, adherence has been found to range between 20 and 81% in groups of adolescents [13, 18]. Hodgkin et al. [19] noted 49% mean adherence in ADHD adolescents, whereas Marcus and Durkin [13] found adherence to stimulant treatment in only one young person out of five as defined by ≥70 in the medication possession ratio (MPR = the number of dispensed medication doses divided by the number of days in a unit of time multiplied by 100).

Medication treatment behaviour may be described in terms of the time on medication or discontinuation, often called persistence [20], or adherence, which captures the extent to which a patient’s actions correspond to the treatment agreement recommendations of the health-care providers [12]. Varying definitions and methodological heterogeneity are responsible for the wide range of outcomes on adherence to ADHD medications. Adherence investigators have used database information on refill intervals or pill counts, patient/care-giver surveys and semi-structured interviews, but there are few studies based on serum concentration measurements [21–24].

Adherence is a multidimensional phenomenon comprising factors related to the patient, condition, therapy, health-care system, and socioeconomic circumstances [12]. Regarding ADHD, factors linked to the patient, therapy, and the condition have been found to affect adherence to pharmacological treatment, including general factors such as culture and body mass index [23], age and gender [24]. One previous study on a group of children and teenagers with ADHD revealed higher adherence among females [24]. Regarding pharmacological factors, non-adherence behaviour has been reported to increase in children and adolescents with a longer period of medication [22, 25]. Side effects were associated with negative feelings towards the ADHD medication and less adherence in an earlier qualitative study [14]. Furthermore, parents of 6 to 18-year-old children with ADHD pinpointed minimal medication effects and side effects as among the most common reasons for discontinuing medication [26]. As many as 29% of children and adolescents report non-serious adverse events of methylphenidate, according to a recent Cochrane review [27], and therefore it appears that side effects are important to take into consideration in adherence studies. The evidence is limited and conflicting regarding the influence of the type of ADHD medication on adherence. Barner et al. [24] found 67% less adherence to immediate-release stimulant treatment compared with non-stimulant treatment in children aged 3–18 years. Wehmeier et al. [25] found similar rates of adverse events and discontinuation of treatment with stimulants versus non-stimulants. Although MPH has been reported to have a higher response rate or efficacy than ATX [28], it is not known how important this factor is in comparison to other factors influencing adherence in adolescents on ADHD medication, especially with regard to the findings of Barner et al. [24]. Hence, the exact relationship of adherence with the benefits and risks of medications seems unclear in young people with ADHD, although this topic has been addressed previously in studies based on parents [29, 30]. However, Ferrin et al. [31] argued that adolescents’ beliefs about the medication have a higher weighting than the real benefits and risks, and this was further highlighted by Charach et al. [32] who found that young persons’ beliefs and attitudes have increasing impact on medication use as the decision-making shifts from the parent to the young person concerned. The belief that medication is effective combined with minimal experience of adverse effects has been shown to increase willingness to use ADHD medication for adolescents [33, 34].

The Beliefs about Medicines Questionnaire (BMQ-Specific) [35, 36] may be used to study the relative impact of beliefs about the benefits versus risks of medication on adherence and is a measure of the necessity-concerns framework (NCF) [37, 38], which is an expansion of Leventhal’s Common-Sense Model (CSM) [39]. The CSM aids the understanding of adaption and management of health and illness threats [40] and constitutes five domains of illness representation: Identity, timeline, cause, consequences and control [41]. Another related and useful questionnaire in adherence investigations is the Brief Illness Perception Questionnaire (B-IPQ), which is also based on the CSM [41] and explores perceptions about the illness. To date, there is only limited information available on perception about ADHD among adolescents, and none regarding possible influences on their adherence to medication. Adolescents have endorsed diverse experiences, such as perceiving ADHD as a chronic physical illness [32], but also positive attributes of having ADHD, or equality with peers [42].

The self-report Medication Adherence Report Scale (MARS) [46] is advantageous in clinical settings because it is short, with only five items. To our knowledge, MARS has not been used to study adherence in ADHD. This is unfortunate, because MARS may contribute to existing knowledge by specifically exploring intentional and unintentional non-adherence [43, 44]. Intentional non-adherence is based on an active decision not to take the medication as prescribed, whereas unintentional non-adherence is due to failure to carry out the intention to take the medication because the medicines are too expensive or due to forgetfulness [43]. To our knowledge, none of the instruments mentioned above have been used for investigating adherence to ADHD medications in adolescents, although they are used in other disorders [37, 45].

The aim was to increase knowledge regarding adherence in adolescents on long-term ADHD medication prescription and in particular the influence of beliefs about the medication and perception of ADHD, in addition to age, time on medication and gender; all of which, we hypothesized would have an impact on adherence.

## Methods

### Procedure

Participants were recruited from two child and adolescent psychiatric clinics (CAP) in Sweden between March 2014 and June 2015. All adolescents (13–17 years) on a long-term prescription of ADHD medication for at least 6 months were consecutively enrolled in the study. Exclusion criteria were autism spectrum disorder, mental retardation (IQ < 70), neurological disorders and language barriers (e.g. inability to answer questionnaires in Swedish). Information about the study was sent with the letter setting up an appointment for ordinary monitoring of prescribed ADHD medication. Written informed consent was obtained both from the adolescent and parent/guardian at the time of the visit. Then, in the presence of the study nurse, the questionnaire was handed out and filled in. In order to get independent results, it was explained that the answers would never reach the doctor or staff responsible for treatment. In addition to socio-demographic questions, the questionnaire included the following self-reports: MARS [46], BMQ-Specific [35, 36] and the B-IPQ [41]. The study was approved by the Regional Ethical Review Board in Linköping (D-nr 2013/402-31).

### Participants

In total, 148 or 92.5% of the 160 patients invited to participate were included and gave written informed consent, of whom 101 or 68.2% (mean age of 15.6 ± 1.4 years), filled in the questionnaires; 66 (66.3%) boys and 35 (34.7%) girls (see flow-chart in supplementary online material). The girls tended to be older (*p* = 0.058) and had a longer pharmacological treatment (*p* = 0.098). The ADHD diagnosis was determined in accordance with DSM-IV criteria by an experienced CAP specialist after a thorough neuropsychological investigation encompassing careful clinical examination, questionnaires and, in most cases, 92% (93/101), also supported by a computer-based assessment of the ADHD core symptoms, the QbTest (Qbtech. Quantitative behaviour technology. https://www.qbtech.com/. Accessed 31 March 2016). All participants had ADHD combined type and had a mean (SD) time on medication of 50.7 (29.3) months (Table 1). Eighty-one of the patients (80.2%) were taking long-acting MPH formulations and 9 (8.9%) ATX only and 11 ATX (10.9%) in combination with MPH (Table 1). In Sweden, MPH is recommended as first-line treatment, whereas lisdexamphetamine was only recently registered, at the time of the study, and immediate release amphetamines could only be prescribed with a special licence and are therefore hardly used. No significant differences regarding gender, age at the start of medication and time on medication were detected in an attrition analysis in which the 47 non-respondents were compared with the 101 participants.

**Table 1:** Group differences (gender, high/low adherence and medication) among adolescents (*n* = 101) on long-term ADHD medication regarding age, medication, adherence by MARS and its subscales, beliefs about prescribed ADHD medication by BMQ-Specific subscales and perception about ADHD by B-IPQ

	Total (*n* = 101) mean (SD)	Boys (*n* = 66) mean (SD)	Girls (*n* = 35) mean (SD)	High adherence (*n* = 47) mean (SD)	Low adherence (*n* = 54) mean (SD)	MPH (*n* = 81) mean (SD)	ATX (*n* = 9) mean (SD)	ATX ± MPH^b^ (*n* = 9 + 11) mean (SD)
Age	15.6 (1.37)	15.4 (1.40)	16.0 (1.28)^a^	15.4 (1.39)	15.9 (1.32)^a^	15.5 (1.41)	16.3 (1.12)^a^	16.2 (1.02)^a^*
MPH	81 (80.2%)	52 (64.2%)	29 (35.8%)	41 (50.6%)	40 (49.4%)	N/A	N/A	N/A
ATX	9 (8.9%)	9 (100%)	0 (0%)	3 (33.3%)	6 (66.7%)	N/A	N/A	N/A
ATX ± MHP^b^	20 (19.8%)	14 (70.0%)	6 (30.0%)	6 (30.0%)	14 (70.0%)^a^	N/A	N/A	N/A
Time on medication	50.7 (29.26)	54.1 (29.46)	44.5 (28.22)^a^	49.8 (27.05)	51.5 (31.24)^a^	49.7 (28.94)	70.6 (29.22)^a^	54.8 (30.93)^a^
MARS total^c^	22.0 (2.25)	22.2 (1.86)	21.7 (2.86)^a^	23.9 (0.70)	20.4 (1.84)^a^	22.2 (2.24)	21.6 (2.19)^a^	21.5 (2.26)^a^
Intentional non-adherence^c^	18.3 (1.86)	18.5 (1.62)	18.0 (2.23)^a^	19.7 (0.59)	17.1 (1.70)^a^	18.3 (1.87)	18.2 (1.86)^a^	18.1 (1.85)^a^
Unintentional non-adherence^c^	3.7 (0.77)	3.7 (0.64)	3.7 (0.98)^a^	4.2 (0.65)	3.4 (0.65)^a^	3.8 (0.76)	3.3 (0.50)^a^*	3.5 (0.76)^a^*
BMQ-Specific–necessity^d^	16.1 (4.53)	15.4 (4.53)	17.4 (4.30)^a^*	17.4 (4.92)	15.0 (3.86)^a^**	16.5 (4.61)	14.2 (4.79)^a^	14.5 (3.85)^a^
BMQ-Specific–concerns^d^	9.3 (3.81)	8.9 (3.57)	10.1 (4.16)^a^	8.1 (3.57)	10.4 (3.71)^a^**	8.8 (3.66)	10.3 (2.35)^a^	11.4 (3.79)^a^**
BMQ-Differential score^d^	6.8 (5.96)	6.5 (5.63)	7.3 (6.60)^a^	9.3 (5.87)	4.6 (5.13)^a^**	7.7 (5.66)	3.9 (5.92)^a^	3.1 (5.80)^a^**
BMQ-side-effects^d^	2.0 (1.18)	1.9 (1.10)	2.1 (1.33)^a^	1.7 (0.99)	2.1 (1.30)^a^	1.9 (1.14)	2.1 (1.05)^a^	2.2 (1.32)^a^
B-IPQ consequence^e^	5.1 (2.43)	4.8 (2.56)	5.6 (2.12)^a^	5.1 (2.65)	5.1 (2.24)^a^	5.2 (2.38)	4.3 (2.92)^a^	4.7 (2.66)^a^
B-IPQ timeline^e^	7.4 (2.59)	7.2 (2.63)	7.8 (2.49)^a^	7.8 (2.36)	7.2 (2.76)^a^	7.6 (2.25)	6.3 (3.71)^a^	6.6 (3.60)^a^
B-IPQ personal control^e^	6.6 (2.10)	7.0 (2.04)	5.9 (2.05)^a^**	7.0 (2.04)	6.2 (2.10)^a^	6.6 (2.07)	7.2 (1.99)^a^	6.5 (2.26)^a^
B-IPQ Treatment Control^e^	7.7 (2.09)	7.5 (2.18)	8.1 (1.90)^a^	8.2 (1.79)	7.3 (2.56)^a^	8.0 (1.84)	5.8 (2.38)^a^**	6.6 (2.69)^a^*
B-IPQ identity^e^	4.6 (2.56)	4.2 (2.64)	5.3 (5.27)^a^	4.6 (2.70)	4.6 (2.46)^a^	4.5 (2.48)	4.6 (2.93)^a^	5.0 (2.89)^a^
B-IPQ concern^e^	2.8 (2.77)	2.3 (2.50)	3.9 (2.97)^a^**	2.9 (2.89)	2.7 (2.69)^a^	2.9 (2.78)	1.8 (2.59)^a^	2.6 (2.80)^a^
B-IPQ comprehensibility^e^	6.7 (5.79)	6.9 (2.48)	6.3 (2.75)^a^	7.2 (2.64)	6.2 (2.45)^a^*	6.7 (2.40)	7.8 (3.11)^a^	6.6 (3.27)^a^
B-IPQ emotional response^e^	5.0 (3.45)	4.4 (3.29)	6.1 (3.53)^a^*	5.2 (3.38)	4.8 (3.53)^a^	5.2 (3.39)	3.56 (3.36)^a^	4.4 (3.68)^a^

*N*/*A* not applicable, *SD* standard deviation

* *p* < 0.05, ** *p* ≤ 0.01

^a^Mann-Whitney’s *U* test vs only MPH

^b^ATX group

^c^Medication Adherent Report Scale

^d^Beliefs about Medication Questionnaire-Specific

^e^The Brief Illness Perception Questionnaire

### Questionnaires

#### Medication Adherence Report Scale

The MARS questionnaire is a self-report scale for assessment of adherence to prescribed medication and consists of five statements, one concerning the subscale of unintentional non-adherence (item 1; “I forgot to take them”) and four concerning the subscale of intentional non-adherence behaviours (item 2; altering the dosage, item 3; stopping taking medication, item 4; missing a dose, and item 5; taking less than instructed) [46]. The items are rated on a 5-point scale, ranging from 1 = very often to 5 = never. Higher scores indicate higher levels of adherent behaviour. Previous studies using dichotomization of MARS conflict as regards a potential correct cut-off point for defining adherence [47–51]. We defined high adherence as ≥92% of total MARS scores (23 of 25). This cut-off point was previously used by another MARS researcher, Mårdby et al. [49], and seemed an appropriate choice in our population in which relative high adherence was expected. In the present study, Cronbach’s alpha for MARS was 0.52.

A separate question “Do you stop taking your ADHD medication during week-ends and school vacations?” was included in the questionnaire (i.e. in addition to MARS) to evaluate how many that sometimes paused their medication as allowed by the doctor.

#### Beliefs about Medicines Questionnaire-Specific

BMQ-Specific has three subscales and eleven questions that capture beliefs about the prescribed medication. The items are rated on a 5-point scale ranging from 1 = “strongly disagree” to 5 = “strongly agree” (overall range, 5–25) [35, 36]. A total score is not calculated as the subscales capture somewhat different dimensions going in opposite directions. Therefore, only the scores of the subscales and the differential are used in statistical analysis. The specific–necessity subscale is based on five questions and investigates beliefs about the necessity of prescribed medication for controlling ADHD symptoms and maintaining health (e.g. “my life would be impossible without my medicines”). The specific–concerns subscale consists of five questions regarding concerns about the negative consequences of taking the medication (e.g. “I sometimes worry about the long-term effect of my ADHD medication”). A necessity–concerns differential score was calculated by subtracting the scores of the specific–concerns scale from those of the specific–necessity scale (range −20 to 20). Therefore, a positive differential score indicates stronger beliefs in the necessity to medicate than concerns about consequences and on the contrary, a negative score indicates stronger concerns. The third subscale, is the one item that involves side effects, “I get unpleasant side effects from my ADHD medicines” [35] and was analysed separately due to reports about side effects of ADHD medication and their known role in non-adherence behaviour [46]. In the present study, Cronbach’s alpha was 0.80 for the specific–necessity scale and 0.75 for the specific–concerns scale.

#### The Brief Illness Perception Questionnaire

The B-IPQ is a 9-item self-report scale that covers respondents’ perceptions regarding ADHD. A total score is not calculated since the questions aim to capture different perceptions, therefore only the scores of the individual items are used in statistical analysis. The first eight items are rated between 0 and 10. Five items assess cognitive dimensions (timeline (chronic vs acute), identity, consequences, and personal and treatment control of ADHD). Two items measure emotional dimensions (concern about ADHD and emotionally affected by ADHD). One item assesses ADHD comprehensibility. A higher score reflects a stronger perception of the respective item regarding ADHD [41]. Item 9 is an open question, which was not included in this analysis.

### Statistical analysis

The Statistical Package for the Social Sciences (SPSS) 21.0 was used. Descriptive statistics, frequencies, means, and standard deviations were calculated. Mann–Whitney’s *U* test was used to compare mean scores between high/low adherence, gender and medication groups. The Chi-squared test was used to compare frequencies between two dichotomized group variables (high/low adherence vs gender). There were too few adolescents using either ATX alone or in combination with MPH, to permit any powerful statistical analysis. Therefore, all users of ATX (with or without MPH) were united and called the “ATX group” to give some information about possible differences in adherence behaviour between ATX and MPH-only consumers. Pearson’s correlation coefficient (95% confidence interval) was used to explore the associations, in the total sample and separately for gender, between scores in the total MARS and its subscales “intentional and unintentional non-adherence” with age, time on medication, “BMQ-specific subscales” and the “BMQ differential score”, as well as the 8 items in B-IPQ. A total score is not used or calculated for the BMQ-specific and B-IPQ scales and is therefore not included in the analyses. Three different stepwise multiple regression models were created in which the scores of (a) the total MARS, (b) intentional and (c) unintentional non-adherence subscales were used as dependent variables [52, 53]. Those variables which had a *p* value less than 0.10 in the prior correlation analyses (Person’s correlation, Table 2) with the total MARS, intentional and unintentional non adherence subscales were included as independent variables. The independent variables included in the model were in (a) and (b); “the BMQ-specific-necessity”, “the BMQ-specific-concerns”, “the BMQ-necessity-concerns differential”, ”the BMQ-unpleasant side-effects” and in model (b) also time on medication, and finally in model (c); “the BMQ-specific-concerns”, “BMQ-necessity-concerns differential” and “B-IPQ-consequence”.

**Table 2:** Correlations of adherence measures by MARS and its subscales with beliefs about medication by BMQ-Specific and perception about ADHD by B-IPQ among adolescents (*n* = 101) on long-term ADHD medication

	Total MARS^a^	Intentional^a^	Unintentional^a^	Age	Time on medication
	*r*	*r*	*r*	*r*	*r*
Age	−0.155	−0.134	−0.131		
Time on medication	0.110	0.171	−0.092	0.278*	
BMQ-Specific-necessity^b^	0.207*	0.186	0.157	−0.048	−0.038
BMQ-Specific-concern^b^	−0.388**	−0.371**	−0.241*	0.171	0.030
BMQ-Differential score^b^	0.406**	0.379**	0.274**	−0.146	−0.048
BMQ-side-effects^b^	−0.281**	−0.288**	−0.127	0.073	0.055
B-IPQ Consequence^c^	0.100	0.020	0.245*	0.188	−0.043
B-IPQ Timeline^c^	0.114	0.072	0.162	−0.063	−0.053
B-IPQ Personal control^c^	0.119	0.130	0.033	0.047	−0.035
B-IPQ Treatment Control^c^	0.107	0.078	0.124	−0.129	−0.162
B-IPQ identity^c^	0.039	0.022	0.060	−0.033	0.004
B-IPQ concern^c^	0.009	0.003	0.019	0.047	−0.015
B-IPQ comprehensibility^c^	0.090	0.066	0.102	−0.025	0.072
B-IPQ emotional response^c^	0.004	−0.044	0.118	−0.030	0.007

*r* Pearson correlation

* *p* < 0.05

** *p* < 0.01

^a^Medication Adherent Report Scale

^b^Beliefs about Medication Questionnaire-Specific

^c^The Brief Illness Perception Questionnaire

## Results

### Adherence

Adherence was measured by MARS, and detailed statistical information is found in Table 1. The mean score for the whole group was 22.0, which is 88% of the maximum score. High adherence behaviour in accordance with our definition (92–100% of maximal MARS scores) was found in 46.5% (*n* = 47) of cases. The total MARS scores showed no difference regarding the time on medication, type of medication treatment, age or gender. Adolescents taking ATX with (*p* = 0.041). or without MPH (*p* = 0.044), compared with those solely on MPH, reported significantly more unintentional non-adherence (Table 1). In total, 50 adolescents (49.5%) endorsed not taking medication during weekends and holidays; 15 always, 11 often, 10 sometimes and 14 rarely.

### Beliefs about ADHD medications

Beliefs about medication, captured by the BMQ, are shown in Table 1. Girls endorsed stronger beliefs than boys regarding the necessity of ADHD medication (*p* = 0.033). The “BMQ necessity–concern differential” scores were positive in the majority of cases or 83.2% (*n* = 84), negative in 11.9% (*n* = 12) and zero in 4.9% (*n* = 5). Participants in the ATX group had significantly more concerns about their treatment (*p* = 0.004) and received lower scores on the “BMQ necessity-concerns differential” (*p* = 0. 004), than those solely on MPH. BMQ ratings were not influenced by age or the time on medication (Table 2).

#### Adherence and beliefs about ADHD medications

**Fig. 1:**
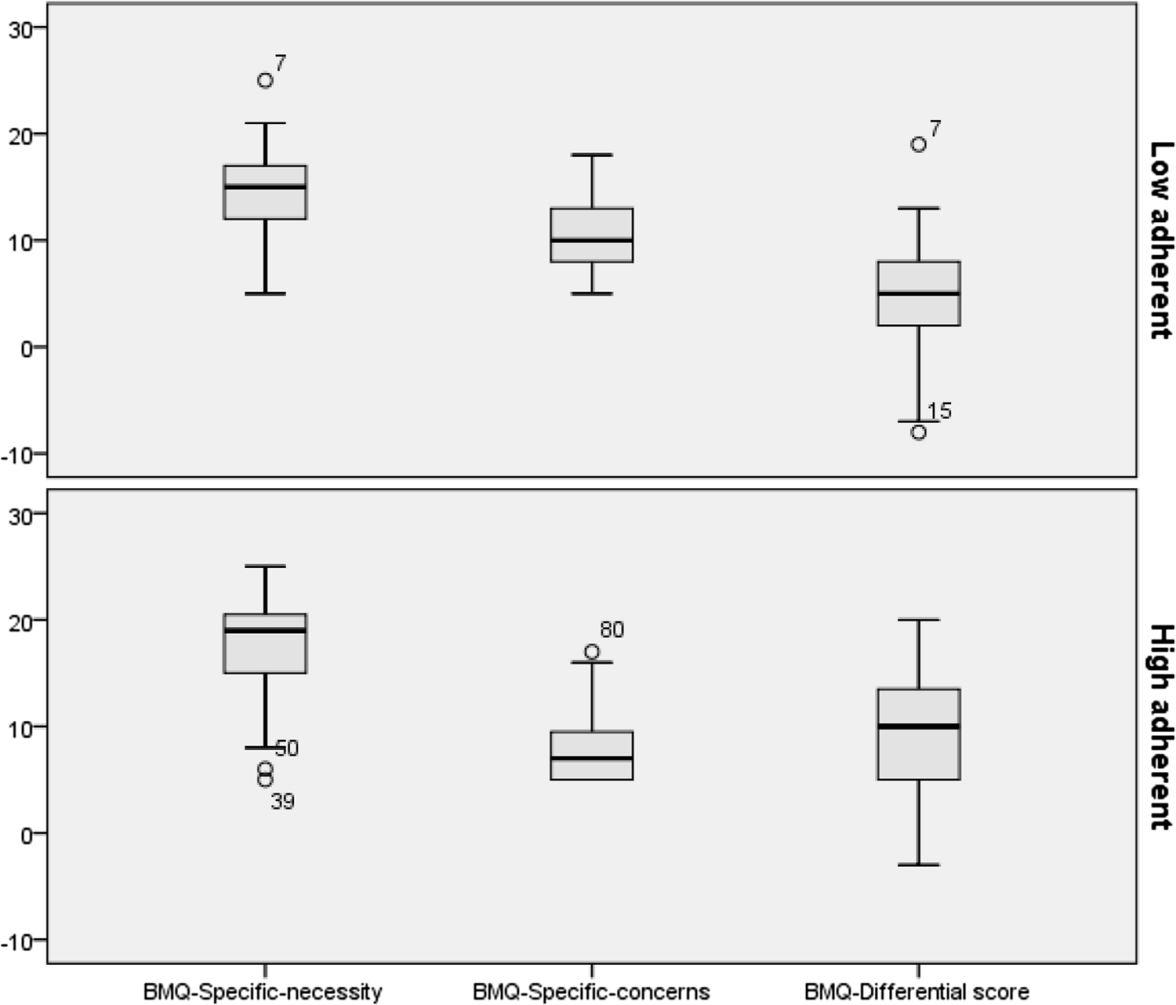
Comparison of medication beliefs among adolescents (*n* = 101) on prescribed ADHD medication divided into low versus high adherence groups

Correlations between MARS and BMQ ratings are shown in Table 2. The scores for the total MARS, intentional and unintentional non-adherence subscales showed statistically significant negative correlations with the scores of the “BMQ, specific–concerns” subscale (*p* < 0.001–0.015) and positive correlations with the necessity–concerns differential score (*p* < 0.001–0.006). The ratings for the total MARS as well as the intentional subscale showed statistically significant negative correlations with the score on the BMQ side effects item (*p* = 0.004 and 0.003, respectively). Findings from the gender specific analyses are shown in Table 3. In girls, the scores for the total MARS, intentional and unintentional non-adherence subscales showed statistically significant positive correlations with the “BMQ, specific–necessity subscale” (*p* = 0.002–0.009), and the “BMQ necessity-concern differential” (*p* < 0.001–0.013). Total MARS and the intentional non-adherence subscale showed negative correlations with the BMQ side effects item (*p* = 0.010 and *p* = 0.007, respectively). For boys, the scores for the total MARS, intentional and unintentional non-adherence subscales showed statistically significant negative correlations with the “BMQ, specific–concerns” subscale *(p* < 0.001–0.008). In addition, the total MARS scores and intentional non-adherence showed a statistically significant positive correlation with the “BMQ necessity-concern differential” (*p* = 0.013 and *p* = 0.018). The group with high as compared with low adherence scored significantly higher on “BMQ necessity” (*p* = 0.002), lower on “BMQ concerns” (*p* < 0.001) and demonstrated a significantly higher “BMQ necessity–concerns differential” score (*p* < 0.001) regarding the ADHD medication (Fig. 1).

**Table 3:** Correlations of adherence measures by MARS and its subscales with beliefs about medication measures according to BMQ-Specific in adolescents (*n* = 101) on long-term ADHD medication shown separately for genders

BMQ variables	Boys (*n* = 66)						Girls (*n* = 35)					
	MARS		Intentional non-adherence		Unintentional non-adherence		MARS		Intentional non-adherence		Unintentional non-adherence	
	*r*	*p*	*r*	*p*	*r*	*p*	*r*	*p*	*r*	*p*	*r*	*p*
BMQ-Specific–necessity	0.043	0.730	0.079	0.527	−0.075	0.552	0.504	0.002	0.434	0.009	0.480	0.003
BMQ-Specific–concerns	−0.424	<0.001	−0.359	0.003	−0.322	0.008	−0.339	0.047	−0.361	0.033	−0.165	0.342
BMQ-Differential score	0.304	0.013	0.291	0.018	0.144	0.248	0.542	<0.001	0.511	0.002	0.418	0.013
BMQ-side-effects	−0.131	0.295	−0.141	0.259	−0.023	0.864	−0.430	0.010	−0.447	0.007	−0.237	0.170

*r* Pearson correlation

### Perception of ADHD

Group differences regarding perception of ADHD between gender and medication groups were analysed with the B-IPQ (Table 1). Boys perceived that they could manage their ADHD (B-IPQ Personal control) better than girls (*p* = 0.005), whereas girls were more emotionally affected by their ADHD disorder (B-IPQ Concern, *p* = 0. 009, B-IPQ Emotional response, *p* = 0.026). The adolescents in the MPH group believed more strongly in the medication´s “controlling effect” of the ADHD symptoms (B-IPQ Treatment Control, *p* = 0.038) than those in the ATX group.

#### Adherence and perception of ADHD

The total MARS scores for the whole group were generally not significantly correlated with the B-IPQ scores, age or time on medication (Table 2). However, the adolescents who felt that ADHD affected their life less (B-IPQ Consequence, *r* = 0.25, *p* = 0.014) had higher ratings of unintentional non-adherence. In girls the scores for the total MARS scale (*r* = 0.37, *p* = 0.027) and the intentional non-adherence subscale (*r* = 0.40, *p* = 0.018) were positively correlated to the B-IPQ question regarding the belief that ADHD duration would be long-standing (B-IPQ Timeline). In boys, only the “unintentional non-adherence subscale” correlated with the B-IPQ question “How well do you feel you understand your ADHD?” (B-IPQ Comprehensibility, *r* = 0.27, *p* = 0.031). The gender analysis is not shown in Table 2.

### Predictive factors for adherence to ADHD medication

In a stepwise multiple regression model (Table 4), two independent variables explained the variance of MARS total scores (*R* ^2^ = 0.21), namely the necessity–concern differential and the BMQ side effects item. For intentional non-adherence (*R* ^2^ = 0.24) time on medication also contributed significantly. For unintentional non-adherence (*R* ^2^ = 0.12) the necessity–concern differential and the B-IPQ Consequence item contributed significantly. A one-unit increase in the necessity–concern differential increased the total MARS score by 0.14 and the intentional non-adherence score by 0.11. A one-unit increase in the “experienced side effects” score decreased the MARS total score by 0.42 and the intentional non-adherence score by 0.38. A one-unit increase in time on medication increased the intentional non-adherence score by 0.01. A one-unit increase in the necessity–concern differential increased the unintentional non-adherence scores by 0.03. A one-unit increase in the B-IPQ consequence increased the unintentional non-adherence score by 0.07.

**Table 4:** The variables significantly explaining the variance of (a) MARS total, (b) the intentional and (c) the unintentional subscales in three corresponding multiple linear regression models in adolescents (*n* = 101) on long-term ADHD medication

Variables	Total MARS			Intentional non-adherence			Unintentional non-adherence		
	B	SE B	*p*	B	SE B	*P*	B	SE B	*p*
BMQ-differential score^a^	0.140	0.034	<0.001	0.110	0.028	<0.001	0.033	0.012	0.009
BMQ-side-effects^a^	−0.424	0.173	0.016	−0.377	0.142	0.009	–	–	–
Time on medication	–	–	–	0.013	0.006	0.027	–	–	–
B-IPQ consequence^b^	–	–	–	–	–	–	0.070	0.030	0.023

*B* regression equation, *SE B* standard error for B

^a^Beliefs about Medication Questionnaire-Specific

^b^The Brief Illness Perception Questionnaire

## Discussion

In a sample of adolescents on long-term ADHD medication, the mean adherence was high or 88% of maximum MARS score, and significantly associated with less experience of side effects and a positive differential of beliefs in the necessity versus concerns of medication. These findings demonstrate that adolescents on long-term medication seem to be able to follow medication prescription despite increasing independence [14]. Age and length of the time on medication were not correlated to MARS total score. Gau et al. [54] found the opposite concerning age. However, they did also find that time on medication had no effect on adherence [54]. Adherence in this study is above average compared to the documented 9.8–71% adherence rates found in earlier studies of combined groups of children and adolescents below 18 years of age, which used similar cut-off points for the definition of adherence or ≥80% cut-off for MPR [15, 24, 55]. For instance, Marcus et al. [13] found 18% high adherence (defined as ≥70% for MPR) in a middle school group, whereas Hodgkin et al. [19] noted 49% mean adherence in adolescents in the same age-span as our population.

There are several methodological discrepancies that may explain the differences in the reported adherence rates, besides potential variations in follow-up routines and socio-demographic factors. The longitudinal nature of previous studies compared with our cross-sectional method may have influenced the results because reliance on memory in cross-sectional studies generates more inaccurate results. We did not replicate the repeatedly reported reduction of adherence with longer time on medication [15, 22, 25], probably due to the cross-sectional design, which may result in participant populations with different medication durations. Bypassing the most critical period for drug adherence in the first 6 months characterized by adverse events and early dropouts, probably also influenced the adherence rate and may underlie the absence of the otherwise anticipated associations between adherence and other factors such as time on medication [15, 56, 57]. Moreover, it could be questioned comparing with populations that also include younger children, as their parents mostly ensure the continuity of the medication intake [22]. Finally, different assessment tools may lead to different adherence prevalence, although no method is recognized as optimal [12, 44] and most of the methods may overestimate compliance [58]. In the population with ADHD, the self-report method has been shown to generate higher prevalence rates than found in more direct approach such as by measurement of saliva concentrations of MPH [21]. Furthermore, MARS captures forgetfulness, dosage alterations, missing doses, and taking less than the dose instructed as a measure of adherence, which are only indirectly comparable with information on refill intervals or pill counts. Bearing the methodological differences described above in mind, our results should be interpreted with caution especially regarding generalizability.

Although, to our knowledge, this is the first time that MARS has been used to investigate adherence in adolescents with ADHD, it has been used in studies of other chronic somatic disorders in the same age group [51]. The adolescents with ADHD exhibited similar or even higher adherence (88%, measured as the percentage of the group’s mean/maximum MARS scores) than both adolescents (81%) [51] and adults (85%) [59] on asthma medication. This could be linked to the ADHD group’s proportionally stronger differential of belief in the necessity of the medication to control ADHD symptoms, relative to the concerns about negative consequences (larger positive differential).

The scores of the total MARS and its two subscales associated positively with the necessity-concern differential irrespective of whether investigated dichotomously (high/low adherence), by correlation or with multiple regression analysis. Around 21% of the MARS total´s variance was explained by the “necessity-concern differential” together with “experienced side effects”. These two latter factors, in addition to time on medication explained 24% of the variance on the “intentional non-adherence” subscale. Finally, around 12% of the variance on the “unintentional non-adherence” subscale was explained by the “necessity-concern differential”, together with the B-IPQ item “consequences of the ADHD”. Taken together, the differential of beliefs in the necessity versus concerns about the medications, “experienced side effects”, time on medication and “perceived consequences of the ADHD” all influenced adherence to medication. These results not only provide evidence for the notion that teenagers weigh up beliefs about benefits versus risks of the medication as considering pros and cons of adherence [31, 32], but they also take into account the consequences of ADHD.

Furthermore, adherence was significantly inversely associated with the BMQ subscales scores of concerns about medication and “endorsed side effects”, according to both correlation and dichotomous analyses in line with previous data [14, 26]. The “concerns” subscale did not, however, survive the stepwise multiple regression analyses, suggesting that it has a less important role amongst other non-adherence risk factors. The finding of an association between adherence and experienced side effects is reasonable in the light of the high frequency of reported non-serious adverse events secondary to stimulant treatment [27], the high discontinuation rate due to adverse effects [56], and the reported relationship between experiences of adverse effects and willingness to use medication [33]. Overall, these data suggest that the BMQ is a useful questionnaire for identification of risk factors of low adherence behaviour when monitoring adolescents on long-term ADHD medication. The “BMQ- necessity-concerns differential” seems to be a robust indicator of adherence; and shows that beliefs about the medication influence adherence in adolescents on long-term ADHD medication. Hence, the present results emphasize the importance of clinicians being on the alert regarding beliefs about ADHD medication and the execution of efficient monitoring of side effects to enable subsequent appropriate actions to minimize them, such as a titration or switch of medication in order to achieve optimal symptoms control and hopefully good adherence [60, 61].

This is the first time, to our knowledge, that unintentional and intentional non-adherence behaviour has been explored for ADHD medication in adolescents. The MARS total and the unintentional and intentional subscales mainly showed the same association patterns with beliefs about medication since they all correlated with “concerns about medication” and the necessity—concerns differential. The MARS subscale “intentional non-adherence” was associated with perceived short ADHD duration and experience of side effects in girls, often coinciding with a brief time on medication, which in turn was also related to “intentional non-adherence”. Hence, “intentional non-adherence” could be reduced by good monitoring of side effects and information about ADHD and medication from the beginning. Regarding respondents’ perceptions about ADHD (according to B-IPQ), the “unintentional non-adherence” subscale was significantly associated with the perception that ADHD only marginally affected their life. In other words forgetfulness could decrease when functional disturbances owing to ADHD become more impeding and the gains from medication more obvious. Such a notion complies with evidence of improved school functioning and quality of life with MPH [62], also recognized by adolescents as improved schoolwork and better peer relationships [34]. Furthermore, the unintentional non-adherence correlated inversely with “understanding of the nature of ADHD disorder” (in boys), pointing to the importance of psycho-education regarding medication and general knowledge about ADHD in adolescents [33].

To our knowledge, this is the first study of gender differences on adherence to ADHD medication in a sample limited to teenagers. No difference regarding adherence according to total MARS scores was detected between the genders, in agreement with a larger epidemiological adherence investigation on several chronic disorders also based on MARS [63]. Nevertheless, some differences emerged in separate gender analyses. MARS and its two subscales associated negatively with “concerns about the medication” in boys, i.e. boys with more concerns tended to be less adherent. In girls, the necessity-concern differential and beliefs in the necessity of medication were associated with higher adherence. The finding of stronger belief in the necessity endorsed by the girls, as compared to boys, is concordant with results from adults with asthma [59] while, on the other hand, the association of concerns with adherence only in boys contradicts results from adults with asthma [64]. This suggests different mechanisms for these associations of beliefs with adherence in these chronic disorders.

To summarize, adherence seems to have more associations with beliefs about the medication in adolescent girls than boys and suggests a need for partly gender-adjusted low-adherence prophylaxis. Adolescent girls may need more accurate dose titration and monitoring to eliminate side effects and information about ADHD progression, whereas the boys need more information about ADHD and medication to alleviate medication concerns. These findings of gender differences in adherence behaviour in young people with ADHD need further research to clarify the exact underpinnings.

Adherence in general was not influenced by whether the medication consisted of ATX (with or without MPH) or only MPH in line with Wehmeier et al.’s [25] findings, but disagreeing with Barner et al. [24]. Yet, the ATX users independent of whether ATX was the sole treatment or used in combination with MPH, showed significantly more unintentional non-adherence and to a lesser extent perceived that their ADHD was controlled by their treatment, than the MPH group. This may relate to the ATX users’s significantly stronger concerns than beliefs in the necessity for medication, and also to their perception of less improvement due to medication, in parallel with results on adults with asthma [65]. In addition, these results may mirror less successful treatment, since ATX is often a second-line medication at the participating and other clinics [66], which in turn may have lower response rate/efficacy compared with MPH [28, 67]. It could be argued that since MPH and ATX pharmacological actions differ, it is unwise to group the ones taking both MPH and ATX together with only ATX users. However, the clinical experience is that patients taking combination therapy of MPH and ATX often claim that they experience less of “on–off” problems after adding ATX. This suggests that the effect of ATX medication is pronounced also in this combination therapy. Although the adolescents on ATX were too few to permit any conclusive statements regarding differences in adherence behaviour between ATX and MPH users, the results seem worth following up with larger replication studies and suggest that ATX users, in particular, may benefit from more support, such as reminder packaging to improve adherence [68, 69].

The present study has some further limitations that should be considered. The decision not to investigate adolescents at the start of medication limits information about initial adherence problems and dropouts. On the other hand, the population was more homogeneous. Consequently, the results can only be interpreted for adolescents on longstanding stable medication. Using MARS as an indirect measure of adherence and not using a direct method (i.e. patient observation or pharmacological analysis) could have inflated results. In order to minimize a self-report bias due to the tendency to give a good impression the questions are worded in a “normalizing” non-adherent behaviour manner [43]. The MARS Cronbach’s alpha was a bit low [70] which along with the fact that the questionnaire only relies on one type of validation, i.e. item inter-correlations [71], constitute two relevant methodological limitations for conclusive interpretation of the adherence results. The fact that one-third of the patients were allowed to take “drug holidays” (i.e. only taking medication on school days) may have interfered with responses to the MARS item “Do you stop taking the medication for some time”, intended to capture non-adherence behaviour. This is suggested by a separate analysis of the 51 individuals who never took any prescribed drug holidays (and could not have misinterpreted that question) where the proportion of high adherents was considerably higher, or 67% as compared to the 46.5% found in the whole group. Future studies are needed for better clarification of how prescribed drug holidays influence adherence measurements and for identifying the most optimal way to handle this dilemma. Furthermore, several factors that might contribute to unintentional non-adherence such as comorbidity and socio-economic status, were not taken into account thereby limiting the generalizability of findings. There may be a risk of alpha errors because of the number of performed analyses but no adjustments were made for mass significance, because of the risk of over-adjusting the significance level due to high correlations between most variables in the domains. Therefore, the results need to be interpreted with caution.

The socio-demographic attrition analyses showed no differences between participants and non-respondents that suggests minimal effects of attrition on the results. The rather high attrition after giving consent is most reasonably explained by a tendency for teenagers and their parents to show cooperativeness when face-to-face with the care-givers, which failed to survive throughout when filling in the questionnaire, especially for those bringing it home, in addition to the fact that many parents of children with ADHD themselves have problems with organization and fulfilling chores. However, it seems likely that the adolescents who chose not to participate or return the questionnaires were less adherent and therefore underestimation of low-adherence behaviour and compromised elucidation of the underlying factors in our data are possible. On the other hand, 69% participation, of all those invited, is acceptable and was possibly due to the well-controlled care including good cooperation with parents and with the research team.

The main strengths of the study are the well-characterized population due to the possibility of identifying all suitable patients undergoing medication at the participating clinics and the foregoing solid diagnostic work including neuropsychological investigation on which the diagnoses were based.

## Conclusions

Adherence was high overall in a well-controlled adolescent population with ADHD undergoing long-term pharmacological treatment and strongly associated with a positive differential of belief in necessity versus concerns of medication and less experienced side effects. Generally, gender differences regarding adherence to ADHD medication were not found. However, adherence in boys was more influenced by concerns about medication and, in girls by beliefs in the necessity of medication and experience of side effects. The results underscore the usefulness of the BMQ questionnaire in identifying beliefs of relevance for adherence, in particular in girls, while information about the perception of ADHD from the B-IPQ seems to have limited value. The results emphasize the importance of careful follow-up routines, including psycho-pedagogical information, partly with a gender-specific focus.

## Electronic supplementary material

Below is the link to the electronic supplementary material.

Supplementary material 1 (DOC 46 kb)
