# Supplementary material for: Beliefs regarding medication and side effects influence treatment adherence in adolescents with attention deficit hyperactivity disorder
Source: Eur Child Adolesc Psychiatry. 2016 Nov 15;26(5):559–71. doi: 10.1007/s00787-016-0919-1 (PMC5394130; doi:10.1007/s00787-016-0919-1)
Supplement: Supplementary file 1 — Supplementary material 1 (DOC 46 kb) [file 787_2016_919_MOESM1_ESM.doc]

**
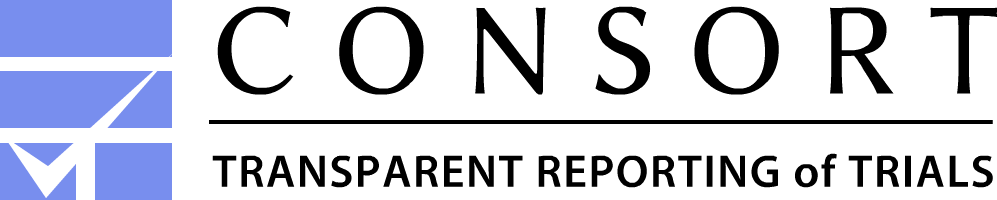
**

**CONSORT 2010 Flow Diagram**

Assessed for eligibility (n=160)

Invited to participate (n=160)

Written informed consent (n=148)

Excluded (n= 12)

♦  Declined to participate (n=12)

Drop-outs (n= 47)

♦  Did not return questionnaires (n=47)

Included

(n=101, 66 boys, 35 girls)
